# Incidence and Prevalence of Childhood Atopic Diseases in Dutch Primary Care

**DOI:** 10.1111/cea.70035

**Published:** 2025-03-14

**Authors:** W. Kuan Chung, Evelien I. T. de Schepper, Laura Struik, Madelon van Tilborg‐den Boeft, Arthur Bohnen, Patrick J. E. Bindels, Evelien R. van Meel

**Affiliations:** ^1^ Department of General Practice Erasmus University Medical Center Rotterdam the Netherlands

**Keywords:** allergic rhinitis, asthma, atopic dermatitis, atopic triad march, epidemiology, family medicine, primary care

**Abbreviations**
95% CI: 95% confidence intervals
AD: atopic dermatitis
AR: allergic rhinoconjunctivitis
AS: asthma
AT: atopic triad
ATC: anatomical therapeutic chemical
GAN: Global Asthma Initiative
GP: general practitioner
ICPC: International Classification for Primary Care
IPCI: Integrated Primary Care Information
IQR: interquartile range
ISAAC: International Study of Asthma and Allergies in Childhood
PM: particulate matter
PYs: patient years
RAST‐test: radioallergosorbent‐test
RPCD: Rijnmond Primary Care

**Summary**

- The prevalence of allergic rhinoconjunctivitis diagnosis and the atopic triad have increased over time.
- Improved recognition of atopic patients in primary care is needed for optimal disease management.

To the Editor,

Atopic diseases, including atopic dermatitis (AD), asthma and allergic rhinoconjunctivitis (AR), are common childhood conditions [1, 2, 3]. Although studies in open populations (via general population‐based surveys) suggest varying prevalence trends, physician‐diagnosed rates in primary care remain underexplored. Recent literature questions the assumption of an increase in atopic disease prevalence, emphasising shifts in increased disease awareness rather than true epidemiological changes [2, 4, 5]. This study examines the prevalence and incidence of atopic diseases in children within the Rijnmond Primary Care Database (RPCD) [6], including children with the atopic triad—that is, children who have been diagnosed with AD, asthma and AR at any time during childhood.

We conducted a retrospective cohort study using RPCD, a regional database covering over 500,000 primary care patients from 240 general practices in Rotterdam–Rijnmond, the Netherlands. We included children aged 0–18 years from 2013 to 2021. Atopic diseases were identified using International Classification for Primary Care (ICPC) codes combined with pharmaceutical prescription data utilising the Anatomical Therapeutic Chemical (ATC) codes. Additional study methods, including the relevant ICPC and ATC codes for case selection, and results are available in the Open Science Framework online repository: https://osf.io/4kwb2/?view_only=959223bea5534b31aef5c84e5ac6c13d.

AD and asthma were defined as at least two consultations and two prescriptions, while AR required two consultations plus either two prescriptions or a positive radioallergosorbent test (RAST). A case was considered resolved if no further ICPC or ATC records appeared for 2 years.

We analysed data from 66,382 children in 2021, of whom 22,123 had AD, asthma or AR. Median follow‐up time was 3.34 years. The incidence rates for AD and asthma remained stable over time, while AR increased from 0.89 to 1.48 per 100 PYs (*p* < 0.001). Corresponding prevalence rates for AR rose from 4.26 to 6.62 per 100 PYs (*p* < 0.001). The prevalence of the atopic triad also increased from 0.14 to 0.28 per 100 PYs (*p* < 0.001). The prevalence rates for all atopic diseases and the age‐specific prevalence rates are displayed in Figure 1.

**FIGURE 1:**
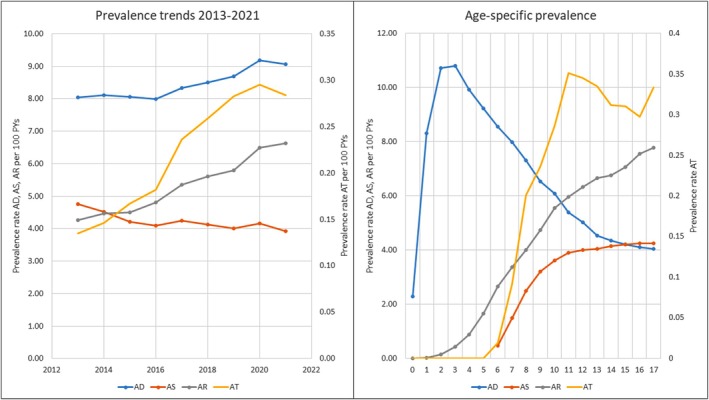
The prevalence rates from 2013 to 2021 and age‐specific prevalence rates for all atopic diseases. In both charts, the *Y*‐axis on the left displays the prevalence rate per 100 PYs for AD, AS and AR, while the *y*‐axis on the right displays the prevalence rate of AT. In the upper panel, the *X*‐axis shows the chronological time in years. In the lower panel, the *X*‐axis shows the patients age in years. AD, Atopic dermatitis; AR, Allergic rhinoconjunctivitis; AS, Asthma; AT, Atopic triad; PYs, Patient years.

Age‐specific analysis (Figure 1) showed peak prevalence for AD at 3 years (median onset: 2.9 years, IQR: 1.0–7.6), asthma at 17 years (median onset: 9.6 years, IQR: 7.6–12.5) and AR at 17 years (median onset: 10.3 years, IQR: 7.3–13.6). The atopic triad peaked at age 11 (0.35 per 100 PYs, 95% CI: 0.28–0.43).

Sex‐specific analysis revealed that AD was more common in boys until age five, after which it became more prevalent in girls. Asthma and AR were more frequent in boys throughout the whole childhood. The atopic triad was more prevalent in boys (0.25 per 100 PYs) than in girls (0.19 per 100 PYs), with peak rates at age 11 for boys (0.41 per 100 PYs, 95% CI: 0.31–0.53) and age 17 for girls (0.34 per 100 PYs, 95% CI: 0.25–0.46).

Our findings indicate stable incidence and prevalence rates for AD and asthma between 2013 and 2021, while AR and the atopic triad prevalence increased. This may be due to previous underdiagnosis, increased physician awareness and environmental factors. Although we did not observe any decrease in prevalence rates during the COVID‐19 pandemic, the impact of COVID‐19 restrictions on healthcare utilisation during 2020–2021 should be considered when interpreting trends. Asthma, AR and AT were more prevalent in boys throughout childhood, whereas AD was initially more common in boys but later in girls after age 13. The atopic triad prevalence was three times higher than expected by chance, supporting findings of distinct atopic disease trajectories. Sex differences align with existing literature, suggesting hormonal and environmental factors contribute to disease persistence [7, 8].

A major strength of our study is the large sample size of the RPCD, which contains data from more than 500,000 primary care patients. However, limitations inherent to database research must be considered. Our study outcomes rely on ICPC and ATC codes, which are subject to individual GP coding variability. While overestimation is minimised by requiring multiple consultations and prescriptions, underestimation may occur in milder cases that require less healthcare. Additionally, disease duration might be underestimated for children with mild symptoms whose cases close after 2 years without follow‐up consultations. Despite this, our estimates align with previous primary care research [9]. Another limitation is the absence of data on ethnicity, family history and environmental exposures such as pets or secondhand smoke. Finally, food allergy could not be studied due to the lack of a specific ICPC code, restricting our focus to the atopic triad of AD, asthma and AR.

In conclusion, the prevalence of AR and the atopic triad is rising in primary care, while AD and asthma did not increase and remained stable. Primary care physicians play a crucial role in identifying atopic children early and ensuring appropriate treatment strategies, including emollient therapy, corticosteroids and lifestyle modifications. Further research is needed to explore risk factors and trajectories in primary care populations.

## Author Contributions

W. Kuan Chung conceptualised this study, performed the extraction, analysis and interpretation of the data and drafted and revised the manuscript. Evelien R. van Meel, Evelien I. T. de Schepper, Madelon van Tilborg‐den Boeft, Arthur Bohnen and Patrick J. E. Bindels were involved in conceptualising the study, data interpretation and critically reviewing the manuscript. Laura Struik performed the data extraction. All authors read and approved the final submitted manuscript and agree to be accountable for all aspects of the work.

## Ethics Statement

The study (project number 2021‐027) was approved by the Governance Board of RPCD.

## Consent

Patient data are de‐identified; therefore, no patient consent was required.

## Conflicts of Interest

The authors declare no conflicts of interest.

## Data Availability

The data that support the findings of this study are available on request from the corresponding author. The data are not publicly available due to privacy or ethical restrictions.
